# Parasites and malignancies, a review, with emphasis on digestive cancer induced by *Cryptosporidium parvum* (Alveolata: Apicomplexa)

**DOI:** 10.1051/parasite/2012192101

**Published:** 2012-05-15

**Authors:** S. Benamrouz, V. Conseil, C. Creusy, E. Calderon, E. Dei-Cas, G. Certad

**Affiliations:** 1 Biologie & Diversité des Pathogènes Eucaryotes Émergents (BDEEP), Centre d’Infection et d’Immunité de Lille (CIIL), INSERM U1019, CNRS UMR8402, EA4547, Université Lille Nord-de-France, Institut Pasteur de Lille France; 2 Environnement et Santé, FLST, Université Catholique de Lille, Université Lille Nord-de-France France; 3 Service d’Anatomie et de Cytologie Pathologiques, Groupe Hospitalier de l’Institut Catholique de Lille France; 4 Instituto de Biomedicina de Sevilla, CIBER de Epidemiología y Salud Pública, Servicio de Medicina Interna, Virgen del Rocío Hospital Universitario Sevilla España; 5 Parasitologie-Mycologie, Centre de Biologie, Pathologie & Génétique, Centre Hospitalier Régional et Universitaire de Lille, Université Lille Nord-de-France France; 6 Cátedra de Parasitología, Escuela de Medicina, “José María Vargas”, Universidad Central de Venezuela (UCV) Caracas Venezuela

**Keywords:** cancer, parasite, *Clonorchis*, *Cryptosporidium*, *Opisthorchis*, protists, *Schistosoma*, infection, cancer, parasite, *Clonorchis*, *Cryptosporidium*, *Opisthorchis*, protistes, *Schistosoma*, infection

## Abstract

The International Agency for Research on Cancer (IARC) identifies ten infectious agents (viruses, bacteria, parasites) able to induce cancer disease in humans. Among parasites, a carcinogenic role is currently recognized to the digenetic trematodes *Schistosoma haematobium*, leading to bladder cancer, and to *Clonorchis sinensis* or *Opisthorchis viverrini*, which cause cholangiocarcinoma. Furthermore, several reports suspected the potential association of other parasitic infections (due to Protozoan or Metazoan parasites) with the development of neoplastic changes in the host tissues. The present work shortly reviewed available data on the involvement of parasites in neoplastic processes in humans or animals, and especially focused on the carcinogenic power of *Cryptosporidium parvum* infection. On the whole, infection seems to play a crucial role in the etiology of cancer.

## Abstract (fr)

Le Centre International de Recherche sur le Cancer (CIRC) recense dix agents infectieux (virus, bactéries, parasites) responsables de pathologies cancéreuses chez l’homme. Parmi les parasites actuellement reconnus comme cancérogènes figurent les trématodes digénétiques *Schistosoma haematobium*, agent de cancer vésical, *Clonorchis sinensis* et *Opisthorchis viverrini*, agents de cholangiocarcinome. Par ailleurs, plusieurs rapports suspectent l’association potentielle entre d’autres infections parasitaires (à protistes ou à métazoaires) et le développement de changements néoplasiques dans les tissues de l’hôte. Cette revue propose une vue d’ensemble des données disponibles sur les relations entre parasites et processus néoplasiques chez l’homme et autres animaux. À la lumière de découvertes récentes, le cas de *Cryptosporidium parvum* fait l’objet d’une analyse plus approfondie. Globalement, les infections semblent jouer un rôle crucial dans l’étiologie du cancer.

## Introduction

Cancer is a leading cause of death worldwide: it accounted for 7.6 million deaths (around 13 % of all deaths) in 2008 (Globocan, 2008). More than 70 % of all cancer deaths occurred in low- and middle-income countries. Deaths from cancer worldwide are projected to continue rising, with an estimated 11 million deaths in 2030 (WHO, 2011).

Cancer is a condition in which abnormal cells divide and grow out of control and are able to invade other tissues. Changes to gene expression patterns are an important feature of cancer cells. These alterations are caused directly or indirectly by genetic or epigenetic events (Stransky *et al.*, 2006). A recent publication of IARC (2011) classified organ, tissue or cell changes associated with cancer in three non-exclusive levels: (a) physiological level, which refers to exposurerelated modifications in the physiology and/or response of cells, tissues and organs (mitogenesis, compensatory cell division, escape from apoptosis, altered cellular adhesion, inflammation, hyperplasia, metaplasia, preneoplasia, angiogenesis, altered steroidal hormone secretion or immunity); (b) cellular level, which refers to exposure-related alterations in the signaling pathways involved in critical cellular processes related to increased risk for cancer (altered expression of genes regulating DNA repair, cyclin-dependent kinases, altered patterns of posttranslational protein modification, changes in apoptosis regulatory factors, in factors involved in DNA replication and transcription or in gap–junction-mediated intercellular communication); (c) molecular level, which refers to exposure-related changes in key cellular structures at the molecular level, including mainly genotoxicity (formation of DNA adducts, DNA strand breaks, gene mutation, chromosomal aberrations, aneuploidy, changes in DNA methylation patterns). These genetic errors which can lead to neoplastic transformation could be caused by hormones, drugs, infectious agents, chemicals, physical or mechanical trauma, and other chronic irritations (Preston-Martin *et al.*, 1990). Among infectious agents, a broad spectrum of them have been linked to cancer, but currently the strength of causal evidence ranges from confirmed, as such Human T cell lymphotropic virus type I (HTLV-I), to speculative, as such *Pneumocystis jirovecii* (de la Horra, 2004).

Around the world, infection is one of the most important causes of cancer and infection-associated cancers are increasing at an alarming rate (De Martel & Franceschi, 2009). The estimated total cancer attributable to infections in the year 2002 was 1.9 million cases, or 17.8 % of the global cancer burden (Parkin, 2006). The main recognized agents are the bacterium *Helicobacter pylori* (5.5 % of all cancer), the human papilloma viruses (5.2 %), the hepatitis B and C viruses (4.9 %), Epstein-Barr virus (EBV) (1 %), human immunodeficiency virus (HIV) together with the human herpes virus 8 (0.9 %) and HTLV-I (0.03 %) (Parkin, 2006). However, other pathogens including parasites can also cause cancer. Among the worms (De Martel & Franceschi, 2009; IARC, 2011), the widespread digenetic trematode *Schistosoma haematobium* can cause urinary bladder cancer and the flukes *Opisthorchis viverrini* and *Clonorchis sinensis* were causally associated with cholangiocarcinoma in extensive areas of the Far East.

Among the parasitic protists, the association of some Apicomplexan and Flagellate species with neoplastic changes in the host tissues was suspected. However, the induction of a host cell transformation was shown experimentally only in the Apicomplexan *Cryptosporidium* and *Theileria*. Actually, it was demonstrated recently that the Apicomplexan *Cryptosporidium parvum* can generate invasive cancer in gastrointestinal and biliary epithelia of SCID mice (Certad *et al.*, 2007, 2010a, 2010b). Regarding *Theileria*, two species *T. parva* and *T. annulata* of cattle were shown to be able of inducing a reversible, parasite-dependent transformation of leukocytes (Dobbelaere & Rottenberg, 2003). Interestingly, many intracellular protists (*Leishmania* spp., *Trypanosoma cruzi*, *C. parvum*, *Toxoplasma gondii*, *Plasmodium* spp., *Theileria* spp.) are known to induce apoptosis inhibition (Carmen & Sinai, 2007), an effect that could be a significant step in the progression to malignancy (Lowe & Lin, 2000).

However, it has been usually difficult to identify pathogens as causative agents of cancers. The usually long latency between primary infection and cancer development is likely one of the main reasons for this remarkable difficulty (zur Hausen, 2009). For instance, the incidence of bilharzian urinary bladder cancer in various African countries peaks between the ages of 40-49 years, while infection with *S. haematobium* begins in childhood (as early as six months of age), and peaked usually in the second decade of life (between the ages of 5-15 years). This data suggest that bladder cancer implies a latency period of 20-30 years to develop from *S. haematobium* infection (IARC, 2011). Sometimes, the geographic coincidence of a specific infection with a defined type of cancer led to reveal a potential causal association. However, in the case of opportunistic pathogens (e.g. *Cryptosporidium* or *Pneumocystis*), it could still be difficult to establish if the pathogen had a causal or a consequential role, even when a specific infection-cancer association was well documented.

At present, infectious agents can be linked to pathology without meeting Koch’s postulates, Hill’s epidemiologic criteria, or even the revised criteria of Hill and Evans. Reproducible epidemiologic and laboratory data are often enough to unambiguously establish that certain infectious agents lead to some chronic outcomes, including cancer. In addition, animal models can often illustrate the plausibility of pathogenesis to humans. Also, clinical trials and surveillance can further demonstrate that preventing or treating the culprit infection avoids or eliminates the effect supporting causality (O’Connor, 2006).

In the present work, we will shortly review available data on the involvement of parasites in neoplastic processes in humans or animals, and we will focus on the carcinogenic power of *C. parvum* infection (Certad *et al.*, 2007; Certad *et al.*, 2010a; Certad *et al.*, 2010b). Indeed, we don’t know if this intracellular protist is able to induce gastro-intestinal or biliary cancer in humans. However, an epidemiological study in Poland reported a high frequency (18 %) of cryptosporidiosis in patients with colorectal cancer (Sulzyc-Bielicka *et al.*, 2007). In short, our experimental observations in the SCID mouse model, the ability of *C. parvum* to inhibit apoptosis in the host cell, and some reports that suggest an association of cryptosporidiosis with cancer in humans, largely justify clinical research aiming at exploring the causal involvement of *C. parvum* in colorectal cancer (CRC) or other digestive cancers in immunocompromised humans.

On the whole, infection seems to play a crucial role in the etiology of cancer. Actually, it was estimated that there would be 26.3 % fewer cancers in developing countries (1.5 million cases per year) and 7.7 % in developed countries (390,000 cases) if cancers associated with infectious diseases were prevented (Parkin, 2006).

## Parasite Protozoa and Cancer

Based on clinical and epidemiological evidences, many reports underlined a potential association between parasitic protozoan infections and cancer. Thus, the flagellate *Trichomonas vaginalis* was suspected to be associated with cervical (Zhang *et al.*, 1995) and prostate cancers (Stark *et al.*, 2009), while the Apicomplexan *Toxoplasma gondii* was suggested to be associated with ocular tumor, meningioma, leukemia and lymphomas (Khurana *et al.*, 2005). It was also suggested that *Plasmodium* could play a co-factor role in the development of Burkitt lymphoma (Khurana *et al.*, 2005). However, only *Theileria* spp. (Dobbelaere *et al.*, 2000; Dobbelaere & Rottenberg, 2003; Lizundia *et al.*, 2006; Branco *et al.*, 2010) and *C. parvum* (Certad *et al.*, 2007; Certad *et al.*, 2010a; Certad *et al.*, 2010b) were clearly shown to be able of inducing a hostcell transformation associated with tumorous disease.

### *Trichomonas Vaginalis* (Excavata: Parabasalia)

*T. vaginalis* is a pathogenic protozoan, sexually transmitted, which resides in the lower female genitourinary tract. *T. vaginalis* coexist frequently with other local infections like pneumocystosis (Duboucher *et al.*, 2003, 2007), candidiasis, bacterial infections, HPV virus infection, the last promoting cervical tumor growth (Boyle *et al.*, 1989). Most serological, cytological and histopathological studies have suggested an association between *T. vaginalis* and the risk of cervical neoplasm (Yap *et al.*, 1995; Sayed el-Ahl et al., 2002; Zhang *et al.*, 1995). But until now, cause and effect relationship has not been proven, and other studies have provided contradictory results that suggest no association between *Trichomonas* infection and cancer (Chakrabarti *et al.*, 1992).

For the last five years, trichomonosis has also been suggested as positively associated with prostate cancer in men, on the basis of the association *T. vaginalis* – seropositive status and prostate cancer risk (Sutcliffe *et al.*, 2006; Stark *et al.*, 2009). This infection seemed to be associated with cancer clinical frames that progress to bony metastasis and prostate cancer death (Stark *et al.*, 2009). The same team developed then another large study (Sutcliffe *et al.*, 2009) in order to test the reproducibility of precedent observations and the consistency of the hypothesis of a trichomonosis-prostate cancer association. However, this second work failed to show an association between *T. vaginalis* serostatus and prostate cancer. Additional work is therefore warranted (Sutcliffe *et al.*, 2009).

### *Theileria* (Alveolata: Apicomplexa)

Intracellular parasites from *Theileria* genus are particularly pathogenic in cattle and lead to a lymphoproliferative disease which is often lethal. *T. parva* and *T. annulata* infections reversibly lead to the transformation of the leukocyte infected cells, which can be reversed using drug that specifically eliminate parasites (Dobbelaere & Rottenberg, 2003). *In vivo*, the host cell transformation is associated with uncontrolled proliferation allowing clonal expansion. *T. parva* infected cells can also get a metastatic phenotype leading to invasion of other host organs (Dobbelaere & Rottenberg, 2003; Lüder *et al.*, 2009).

Leukocyte metabolic pathway and molecular alterations have been investigated during *Theileria* infection and it has been established that multiple host-cell pathways are altered (Table I). Firstly, the anti-apoptosis signaling pathway is stimulated by the activation of the transcription factor NF-κB (Heussler *et al.*, 2002). Secondly, the cytokine GM-CSF (= granulocyte macrophage colony stimulating factor) secretion is enhanced and itself re-stimulate infected host cell proliferation via autocrine loops. Thirdly, GM-CSF contributes to the induction of the factor c-Myc, leading to lymphocyte proliferation (Baumgartner *et al.*, 2000; Dessauge *et al.*, 2005). Endly, *Theileria* dependent transformation leads to the constitutive activation of c-jun kinase (JNK) and permanent induction of activator protein 1 (AP-1) (Lizundia *et al.*, 2006).

**Table I.** Molecular factors or mechanisms linking parasite-related inflammation with cell transformation or cancer.

Parasite	Pathology in natural (N) or experimental (E) hosts	Activated host transcription factors	Inflammatory cytokines	Procarcinogens or mediators of oxidative response	Change in coding or expression of proteins involved in regulating cell cycle or apoptosis	DNA damage or cytoskeleton alteration
Protozoa						
*Cryptosporidium parvum*	Digestive carcinoma (E)	NF-κB (5)	IL-6 IL-8 (13, 17)	–	c-Myc, Bcl2 (15)	Host actin remodeling (23)
			IFN-γ (30)		Cyclin D1 (6)	
			PGE2 (14)			
			TNF-α (1)			
*Theileria* spp.	Lympho-proliferation (N, E)	NF-κB (10)	GM-CSF (2)	–	c-Myc (7)	Cytoskeleton modification (3, 26, 27)
		JNK (8)			c-Jun (15)	
		AP-1 (16)			NF-κB (10, 26)	
		STAT 3 (7)				
Helminthes						
*Clonorchis sinensis*	Cholangiocarcinoma (N, E)	E2F1 (12)	–	–	Cyclin B (12)	DNA oxidation (31)
*Opisthorchis viverrini*	Cholangiocarcinoma (N, E)	NF-κB (22)	IL-6, IL-8 (20, 29) IL-12 (11)	NO (21, 28)	–	DNA oxidation (20, 29)
*Schistosoma haematobium*	Bladder carcinoma (N, E)	–	TNF-α (24)	Aflatoxins (25) ROS, NO (19, 25) *N*-nitroso compounds (18)	p53 (17) Bcl2 (4) COX-2 (9)	DNA alkylation (31)

(1) Barakat *et al.* (2009);

(2) Baumgartner *et al.* (2000);

(3) Baumgartner (2011a);

(4) Chaudhary *et al.* (1997);

(5) Chen *et al.* (2005);

(6) Deng *et al.* (2004);

(7) Dessauge *et al.* (2005);

(8) Dobbelaere et al. (1999);

(9) El-Sheikh *et al.* (2001);

(10) Heussler *et al.* (2002);

(11) Jittimanee *et al.* (2007);

(12) Kim *et al.* (2008);

(13) Laurent *et al.* (1997);

(14) Laurent *et al.* (1998);

(15) Liu *et al.* (2009);

(16) Lizundia *et al.* (2006);

(17) Maillot *et al.* (2000);

(18) Mohsen *et al.* (1999);

(19) Mostafa *et al.* (1994);

(20) Ninlawan *et al.* (2010);

(21) Pinlaor *et al.* (2004);

(22) Pinlaor *et al.* (2005);

(23) Plattner & Soldati-Favre (2008);

(24) Raziuddin *et al.* (1993);

(25) Rosin et al. (1994a);

(26) Schmuckli-Maurer *et al*. (2010);

(27) Schneider *et al.* (2007);

(28) Sripa *et al.* (2007);

(29) Sripa *et al.* (2009);

(30) Stephens *et al.* (1999);

(31) Vennervald & Polman (2009)

*Theileria* intracellular microorganisms reside freely in the host leukocyte in direct contact with host-cell cytoplasmic structures, and modify host-cell cytoskeleton. Parasites interact likely with host-cell microtubules during mitosis to ensure its own persistence and survival (von Schubert *et al.*, 2010). Furthermore, *Theileria* alters host-cell actin dynamics, increases motility and enables infected host-cell to behave as leukocyte metastasis (Baumgartner, 2011a, b).

### *Plasmodium* Species and *Toxoplasma Gondii* (Alveolata: Apicomplexa)

Malaria has been suggested as a co-factor for the development of endemic Burkitt Lymphoma (eBL) that is one of the three clinical variants of this non-Hodgkin lymphoma. It is a B-cell tumorous disease characterized by a chromosomal translocation that results in deregulation of the c-Myc oncogene (Orem *et al.*, 2007). The hypothesis of a potential role of malaria as co-factor for eBL has been supported by several epidemiological studies showing that eBL is more frequent in areas where malaria is endemic. Consistently, a recent case-control study showed a strong significant association between eBL and malaria antibodies (Carpenter *et al.*, 2008). According to the same case-control study, malaria and Epstein Barr Virus (EBV) could act synergistically in the development of eBL. Indeed, malaria, like HIV-infection, induces polyclonal B-cell activation and hypergammaglobulinemia possibly through the stimulus by a superantigen (Bornkamm, 2009). Actually, the cysteine-rich interdomain region 1alpha (CIDR1alpha) of *P. falciparum* erythrocyte membrane protein 1 (PfEMP1), expressed in red blood cells, was shown to act as both a polyclonal B cell activator and an inducer of EBV lytic cycle (Chêne *et al.*, 2007; Bornkamm, 2009). However, more work is needed to clarify the etiology of eBL as even the molecular contribution of EBV to the pathogenesis of BL remains unclear.

The association of *Toxoplasma* infection and adenomas has been suspected in two case reports of pituitary adenoma (Zhang *et al.*, 2002). Adenomas were found associated with *T. gondii* cysts among the tumor cells in patients without immunesuppression status. Potential relations between *T. gondii* and tumors have also been reported in ocular tumors, meningioma, leukaemia and lymphoma (Khurana *et al.*, 2005). The role of *T. gondii* remains to be explored. Interestingly, *T. gondii* was shown to be able to induce increased host-cell (dendritic cells and macrophages) motility, an effect that could favor both *in vivo* spread of parasites and progression of tumorous disease (see the recent review of Baumgartner, 2011b). Paradoxically, *T. gondii* could inhibit tumor growth in the Lewis lung carcinoma mouse model through the induction of Th1 immune responses and antiangiogenic activity (Kim *et al.*, 2007).

### The Special Case of *Cryptosporidium* (Alveolata: Apicomplexa)

Cryptosporidiosis represents a major public health problem. This infection, caused by an Apicomplexan Protozoa of the genus *Cryptosporidium*, has been reported worldwide as a frequent cause of diarrhea, and its prevalence varies according to different regions (Xiao, 2010). In developed countries, massive *Cryptosporidium* foodborne and waterborne outbreaks are quite frequently reported. In developing countries, besides outbreaks, sporadic cases of cryptosporidiosis are frequent, this infection affecting mostly children under five (Xiao *et al.*, 2004). Furthermore, cryptosporidiosis remains a clinically significant opportunistic infection in immunocompromised patients, causing potentially life-threatening diarrhea, especially in those HIV-infected subjects without access to highly active antiretroviral therapy (HAART) (Pozio & Morales, 2005). Additionally, *Cryptosporidium* species not only infect humans, but also cause morbidity in farm animals, leading to economic losses (Sunnotel *et al.*, 2006).

**Fig. 1.**
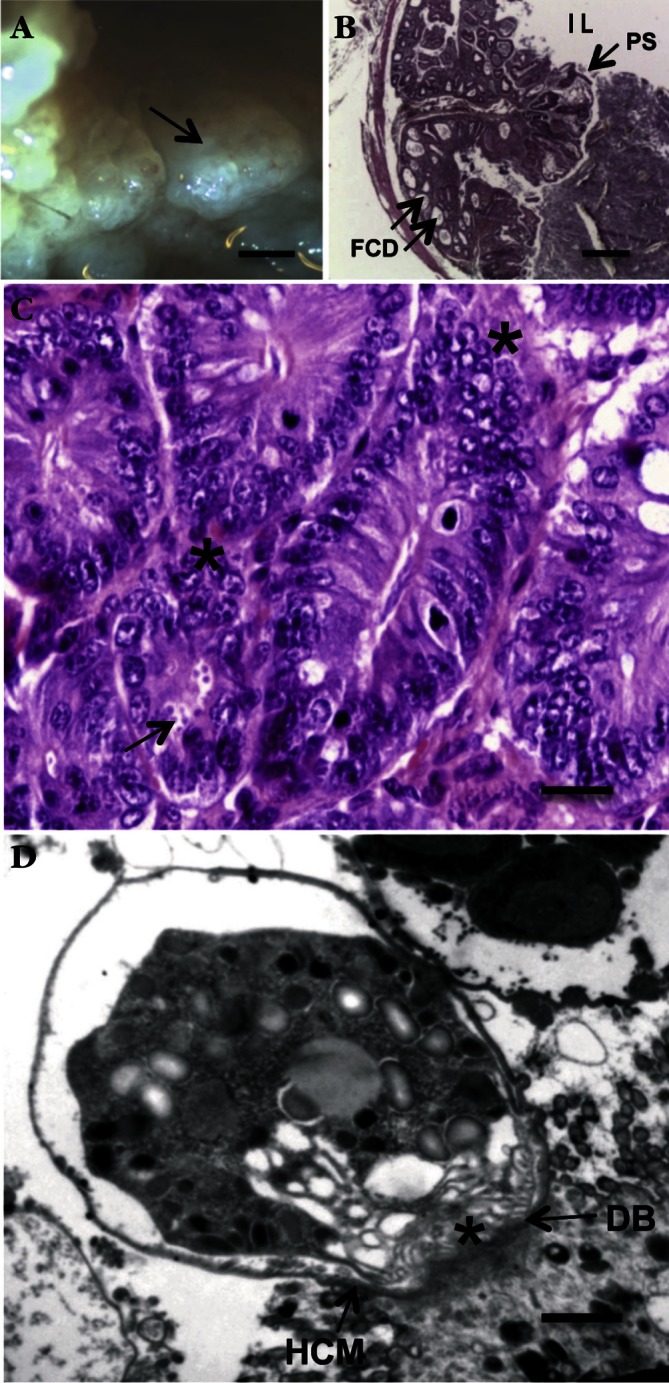
Ileocaecal adenocarcinoma induced by *Cryptosporidium parvum* in dexamethasone-treated SCID mice. A: adenomatous masses (arrow) in the intestinal lumen. SCID mouse orally infected with *C. parvum* oocysts and euthanatized 45 days post-infection; B: projection of a polypoid structure (PS) with focal cystic dilations (FCD) developing inside the intestinal lumen (IL). Hematoxylin & Eosin staining; C: high grade epithelial neoplasia characterized by architectural distortion and cellular atypias (*): loss of normal polarity, nuclear stratification and prominent nucleoli, associated to the presence of numerous parasites inside the glands at the surface of epithelium (arrow). Dexamethasonetreated SCID mouse infected by *C. parvum* after 100 days postinfection. Hematoxylin & Eosin staining; D: transmission electron micrograph showing intracellular *C. parvum* developmental stages inside their parasitophorous vacuole. A feeder organelle (*) adheres to the host cell membrane (HCM) on an electron-dense area, the dense band (DB). Bar (μm) = A: 1,000; B: 400; C: 25; D: 0.4.

*Cryptosporidium* organisms reside in an intracellular parasitophorous vacuole placed at the apical site of gastrointestinal epithelial cells (Plattner & Soldati- Favre, 2008). This unusual location (Fig. 1) was currently qualified as “extracytoplasmic” (Chalmers & Davis, 2010) though others qualified it as “epicellular” (Valigurova *et al.*, 2008). Infection of the intestinal cells can result in blunting of the intestinal villi, crypt hyperplasia and inflammation. Most *Cryptosporidium* species infect the epithelium of the gut but in severe infections, dissemination can occur to extra-intestinal sites (Lopez-Velez *et al.*, 1995).

There were some speculations about possible associations between *Cryptosporidium* infection and neoplasia in humans. The association of cryptosporidiosis and colonic adenocarcinoma was suspected in the case of a Spanish patient carrying both, who died rapidly after the onset of symptoms (Izquierdo *et al.*, 1988). A cryptosporidiosis case of the biliary tract clinically mimicking a pancreatic cancer in an AIDS patient was also reported (de Souza Ldo et al., 2004). Furthermore, an association between X linked immunodeficiency with hyper IgM syndrome (XHIM), and carcinoma of the liver, pancreas and biliary tree was described in children by Hayward *et al.* (1997). These authors proposed that the mutation responsible for this defect may favor the colonization of the biliary epithelium by different pathogens, including *Cryptosporidium*. A chronic infection and inflammation by this parasite will follow, perhaps being the inflammation the cause of the malignant transformation (Hayward *et al.*, 1997).

More recently, an epidemiological study in Poland reported 18 % of cryptosporidiosis in 55 patients with colorectal cancer (Sulzyc-Bielicka *et al.*, 2007) being this prevalence higher compared with the burden of cryptosporidiosis for the general European population (Semenza & Nichols, 2007). However, an unexpected high prevalence of *Cryptosporidium* infection was recently reported in hospitalized patients in Poland (Bajer *et al.*, 2008). Actually, nested PCR revealed a global prevalence of 34 % in 35 patients from three Poland hospitals. In this study, prevalence was 43 % in patients apparently immunocompetent (6/14 patients), 29 % in patients with primary immunodeficiency (5/17 patients) and 25 % in patients with secondary immunodeficiency (1/4 patients) (Bajer *et al.*, 2008).

Furthermore, previous works had associated cryptosporidiosis with the development of benign tumors in naturally or experimentally infected vertebrates. Thus, *Cryptosporidium* organisms were detected in aural-pharyngeal polyps in naturally infected iguanas by using optical or transmission electron microscopy (Fitzgerald *et al.*, 1998; Uhl *et al.*, 2001). Another study reported the presence of *Cryptosporidium* organisms in intestinal polyps of naturally infected sheep (Gregory *et al.*, 1987). *C. parvum* organisms were found to be associated with cystic hyperplasia of the colonic mucosa in experimentally infected nude mice (Heine *et al.*, 1984). None of these studies described the presence of malignant or even pre-malignant lesions.

Nevertheless, the occurrence of dysplastic changes classified as low grade intraepithelial neoplasia of bile ducts was previously reported in a model of IFN-γ knockout mice infected with *C. parvum* (Stephens *et al.*, 1999). The authors of this study suggested that the response of these mice to *Cryptosporidium* infection may model the initial steps towards the development of cholangitis and bile duct-related cancer often observed in patients with immunodeficiency (Stephens *et al.*, 1999). These findings were consistent with the observations of Hayward *et al.* (mentioned above), in relation to XHIM (Hayward *et al.*, 1997).

More recently, the ability of *C. parvum* to induce gastrointestinal neoplastic changes was established experimentally in severe combined immunodeficiency (SCID) mice (Certad *et al.*, 2007; Certad *et al.*, 2010a; Certad *et al.*, 2010b). Herein, adenomas with low or high-grade intraepithelial, intramucosal or invasive neoplasia associated with numerous *C. parvum* life stages were detected in different areas of the digestive tract (Fig. 1) including stomach, duodenum and ileocaecal region. The first time, such lesions were observed unexpectedly after 46 days post-infection (Certad *et al.*, 2007) in *C. parvum* (Iowa strain) chronically infected SCID mice submitted to oral dexamethasone (Dex) administration. These findings were recorded using inoculum between 105 and 108 oocysts. The use of Ki-67 supported the neoplastic nature of the described *Cryptosporidium*-induced epithelial transformation (Certad *et al.*, 2010a), and showed that potential neoplastic alterations begin before the detecting of histopathological lesions by using standard stains.

A highly significant correlation was found between the extension of cryptosporidiosis and the severity of neoplastic lesions in either Dex-treated or untreated SCID mice (Certad *et al.*, 2010a). The ability of other *C. parvum* strains to induce digestive neoplasia in Dextreated SCID mice was also explored. Thus, SCID mice infected with *C. parvum* TUM1 strain developed a fulminant cryptosporidiosis associated with intramucosal adenocarcinoma, which is considered an early histological sign of invasive cancer (Certad *et al.*, 2010b). Thus, the ability of *C. parvum* to induce neoplasia was not restricted to a specific *C. parvum* strain. In contrast, the species *C. muris* revealed unable to induce neoplastic changes in the SCID mouse model (Certad *et al.*, 2007). Reasons of this different expression of the disease according to *Cryptosporidium* species are unclear, though variability in pathogenicity according to *Cryptosporidium* species, strains, and likely to other factors, were already reported (Okhuysen & Chappell, 2002).

No data about the mechanism of *C. parvum*-induced neoplasia are available. Several observations in our study suggest that combination of *C. parvum* with Dex administration is involved in the generation of these significant histological changes in digestive epithelia of SCID mice. This steroid potentially alters innate immunity (Franchimont, 2004; Stojadinovic *et al.*, 2007) in *C. parvum* infected animals. Consistently, a higher *C. parvum* oocyst shedding was found in Dex-treated SCID mice presenting neoplastic lesions (Certad *et al.*, 2010a). However, even lesions with low grade dysplasia induced by *C. parvum* should be considered as a putative precursor to digestive carcinoma. Actually, colorectal tumorigenesis is believed to involve sequential genetic changes leading to both initiation of the neoplastic process and progression from normal epithelium to carcinoma (Luperchio & Schauer, 2001). Taken together, higher rates of oocyst shedding seemed to be associated with an earlier onset and more rapid evolution of neoplastic lesions (Certad *et al.*, 2010a). SCID mice infected with lower inoculum could reach similar rates of both oocyst shedding and severity of lesions, but they may require a longer period (Benamrouz *et al.*, 2011, unpublished; Certad *et al.*, 2010b).

Recent studies have reported about the capacity of some protozoa to interfere with signaling pathways of the host cell (Heussler *et al.*, 2001; Carmen & Sinai, 2007) (Table I). It is well known that *T. parva*, another Apicomplexan protist, is responsible for a lymphoproliferative disorder of cattle (Dobbelaere *et al.*, 2000) (see above). This organism infects and transforms bovine lymphocytes resulting in tumors with metastatic/invasive potential by different mechanisms: (a) inhibition of apoptosis that induces host cell survival; (b) induction of proliferation of the host cell; (c) induction of leukocyte survival by apoptosis inhibiting (Lüder *et al.*, 2009).

Interestingly, inhibition of apoptosis (Table I) was also reported in other Apicomplexan Protozoa including *Cryptosporidium* (Heussler *et al.*, 2001; Carmen & Sinai, 2007). Using an *in vitro* model of biliary cryptosporidiosis, *C. parvum* was shown to be able of activating NF-κB pathway in directly infected cells, preventing the induction of cell death after infection (Chen *et al.*, 2005). NF-κB family of transcription factors regulates the activation of a number of intracellular survival signals including the c-Myc protooncogene (Chen *et al.*, 2001). Indeed, activation of NF-κB has been observed in many cancers, including colon cancer (Naugler & Karin, 2008). The delay in the activation of NF-κB suggests that *C. parvum*-induced NF-κB activation is not directly associated to the attachment and invasion of the cell by the parasite (Chen *et al.*, 2001). However, it has been found in *in vitro* studies that *C. parvum*, depending on its developmental stage, not only inhibits but it can modulate host-cell apoptosis, inhibiting apoptosis at the trophozoite stage and promoting this process at the sporozoite and merozoite stages (Mele *et al.*, 2004). Modulation of apoptotic pathways was also investigated by microarray analysis in an *in vitro* model using human ileocaecal HCT8 cells. Genome wide expression profiling revealed high proportion of apoptosis genes regulated during *C. parvum* infection (Liu *et al.*, 2009). Analysis of the apoptosis gene transcript profile suggests a biphasic control of host cell apoptosis. BCL2 and c-Myc genes showed an altered expression during *C. parvum* infection in HCT8 cells (Liu *et al.*, 2009). Apoptosis prevention probably benefits the parasite by stabilizing the host cell long enough to permit the completion of the life cycle (Heussler *et al.*, 2001). Indeed, resistance to apoptosis could be an essential step in the progression to malignancy (Lowe & Lin, 2000).

Interestingly, it is well known that the parasite induces modifications of the host actin cytoskeleton of intestinal epithelial cells (Table I) though little information is available about the significance of the host actin remodeling process (Plattner & Soldati-Favre, 2008). It has been reported, however, that intercellular and cell matrix adhesion molecules are involved in regulation of cell polarity, differentiation, proliferation and migration. Particularly, in colorectal cancer, cells lose actin cytoskeletal organization and normal cell adhesion when they become invasive (Buda & Pignatelli, 2004). Consistently, alterations in host cell cytoskeleton have been described as a consequence of *Theileria* infection (Lüder *et al.*, 2009) (see above). Therefore, persistent infection with *C. parvum* could be a risk for gastrointestinal neoplasia. Unfortunately, understanding the biology of this parasite has been hampered by the lack of transfection techniques and to the absence of reliable *in vitro* models.

Research on the topic could be worthwhile since the incidence of *Cryptosporidium* infection seems to increase not only amongst immunosuppressed patients but also in the general population worldwide. In the United States a dramatic increase in the incidence of cryptosporidiosis in recent years was reported, mainly due to a substantial rise in community outbreaks (Yoder & Beach, 2010). Moreover, low infectious threshold and the chlorine resistance of oocysts make *Cryptosporidium* ideally suited for transmission through drinking and recreational water, food, person to person and animal to person contact, facilitating the expansion of the infection in the community (Yoder & Beach, 2010).

## The Worms That Induce Cancer in Humans

The causal involvement of parasitic worms in human cancer was firstly suspected on the basis of epidemiological data, especially on the geographic coincidence of a specific infection with a particular type of cancer. At present, IARC (2011) classified *S. haematobium*, *C. sinensis* and *O. viverrini* in group 1 (infectious agents definitely “carcinogenic to humans”), while *Schistosoma japonicum* was classed in the group 2B (agents “probably carcinogenic to humans”) and *Schistosoma mansoni* and *Opisthorchis felineus* were assigned to the group 3 (agents “not classifiable as to its carcinogenicity to humans”).

### *Schistosoma Haematobium* (Digenea: Schistosomatidae)

*S. haematobium* is usually recognized as a definite cause of urinary bladder cancer (Creusy *et al.*, 2010). The relative risk for bladder cancer in *S. haematobium* infected patients has been estimated between 1.8 and 23.5 (Parkin, 2006). About 3 % of urinary bladder cancers around the world are attributable to *S. haematobium* (de Martel & Franceschi 2009).

Digenea trematodes of *Schistosoma* genus belong to Schistosomatidae, the sole trematode family whose members are gonochoric worms (with separated sexes). *S. haematobium* adult worms dwell inside blood vessels of the perivesical venous plexus. As in other schistosomes, the body of *S. haematobium* male worm forms a groove or gynaecophoric channel, in which it permanently holds the thinner and longer female. The *S. haematobium* female produces hundreds of eggs per day throughout her life (usually three-five years). Successive reinfections are usual in endemic areas, where the prevalence of urinary schistosomiasis, especially in children, can be very high. The eggs (145 μm × 60 μm) with a typical terminal spine pass through the walls of the blood vessels, and through the ureteral or bladder wall to be excreted with urine. However, about half of the released eggs do not reach the bladder lumen and are carried back into the liver by the bloodstream and/or trapped in the bladder wall tissues. Retained eggs provoke a granulomatous inflammatory response that enhances local tissue ulceration, being the main cause of disease in the human host. Additionally, eggs provoke pseudopolyposis of the bladder and ureteral walls; chronic lesions can then evolve into fibrosis and carcinoma (de Martel & Franceschi, 2009).

Frequently associated with inflammatory response provoked by the eggs there is conversion of the transitional epithelium to a squamous metaplastic epithelium, which has a much greater proliferative potential (Abol-Enein, 2008). As a consequence, most people with chronic schistosomiasis and bladder cancer develop squamous cell carcinoma (SCC) rather than transitional cell carcinoma (TCC) (Cohen et al., 1992). Consistently, high prevalence of SCC of bladder was observed in geographic areas where *S. haematobium* is endemic (e.g. Egypt, Iraq, Zambia, Malawi, Kuwait) (Mostafa *et al.*, 1999). The carcinogenic role of *S. haematobium* was also supported by several case-control studies and by experimental studies in animals (sees for review Vennervald & Polman, 2009).

Regarding mechanisms, several studies indicate that the carcinogenic power of *S. haematobium* is a multifactorial and multistage process (IARC, 2011) where several processes are involved. Many reviews were devoted to the topic for the last years (Rosin et al., 1994a, Rosin et al., 1994b; Mostafa *et al.*, 1999; Herrera & Ostrosky- Wegman, 2001; Mayer & Fried, 2007; Vennervald & Polman, 2009). Main data may be summarized as follows (see also Table I):

1. Local inflammation, regenerative process and increased cell proliferation (Cohen *et al.*, 1990, 1991) over long periods could provide more opportunities for the expression of spontaneous DNA alterations (Abol-Enein, 2008). Consistently, *S. haematobium* infection was found to be strongly associated with an increased risk of metaplasia or hyperkeratosis. Actually, in endemic areas, prevalence of inflammation (39 %), hyperkeratosis (30 %), metaplasia (33 %) and frank atypia (0.4 %) were much higher than in nonendemic ones (IARC, 2011).
2. Schistosomiasis-induced inflammatory cells have been shown to participate in the metabolic activation of procarcinogens, such as aflatoxins, and in the formation of carcinogenic nitrosamines from nitric oxide (Rosin et al., 1994a; Mostafa *et al.*, 1994). In addition, the often associated chronic bacteriuria produces also nitrosamines, including N-butyl-N-(4-hydroxybutyl) nitrosamine, a known bladder carcinogen in rodents and dogs (Hicks *et al.*, 1982; El-Bolkainy, 1983). What’s more, urinary stasis due to *S. haematobium* infection further contribute to locally concentrate nitrosamines and other endogenous carcinogens, favoring their absorption from urine to the bladder epithelium (Bhagwandeen, 1976). Nitrosamine was apparently an important factor to the development of neoplastic changes in baboons experimentally infected with *S. haematobium*, though alone, nitrosamine was unable to induce neoplasia (Hicks *et al.*, 1980; IARC, 2011).
3. The inflammatory response around the eggs generates genotoxic factors and products that may cause genomic instability as well as stimulate a proliferative response of the host cells to repair tissue damage caused by the inflammation. Reactive oxygen species (ROS) have a role in the etiology of cancer, and inflammatory cells, such as eosinophils, which are an important endogenous source of oxygen radicals, are found in high numbers in urine from people infected with *S. haematobium* (many sources, see Vennervald & Polman, 2009, for review). Consistently, levels of 8-hydroxy-2’-deoxyguanosine (8-OHdG), a critical biomarker of oxidative stress and carcinogenesis, were markedly increased in urinary bladder cell carcinomas associated with schistosomiasis when compared to non-schistosomiasis-associated cancers. DNA-repair genes 8-oxyguanine-DNA-glycosylase and apurinic/ apyrimidinic endonuclease were strongly overexpressed.
4. Also, increased formation of DNA single strand breaks, due to oxidative damage, and higher inducible nitric oxide synthase was found in bladder SCC-associated with schistosomiasis when comparing with non-schistosomiasis-associated cancers (Salim *et al.*, 2008).
5. Genetic instability of host cells may lead to modifications in the regulation of tumor-suppressor genes and oncogenes interfering with the control of cell proliferation (Vennervald & Polman, 2009). Thus, mutations in the tumour-suppressor gene p53 have been observed more frequently in patients with schistosomiasis- associated bladder cancer than in patients with bladder cancer not related to schistosomiasis (Mostafa *et al.*, 1999). Elevated levels of expression of oncoproteins, such as Bcl-2 (Chaudhary *et al.*, 1997; Swellam *et al.*, 2004) and p53 (Chaudhary *et al.*, 1997) have also been observed in schistosomiasis-associated bladder cancer (Vennervald & Polman, 2009).
6. DNA alkylation damage indicated by increased levels of O6-methyldeoxyguanosine (O6-Me-dG) has been demonstrated in tissue samples from schistosomiasis- associated bladder cancer patients. Consistently, O6-alkylguanine-DNA alkyltransferase (ATase) activity was significantly higher in normal bladders than in bladders with cancers and negatively correlated with the levels of O6-Me-dG (Badawi *et al.*, 1992, 1994).
7. Schistosomiasis-associated urinary bladder cancer samples had more genes methylated than non-schistosomal bladder cancer samples in a study in Egyptian patients (Gutiérrez *et al.*, 2004).
8. Although biomarkers may not indicate overt cancer (Vennervald & Polman, 2009), the results from a study in Ghana showed a close correlation between BLCA-4 (a nuclear matrix protein involved in gene regulation and produced only in neoplastic cells), quantitative nuclear grading (QNG) and severe urinary bladder damage such as bladder wall masses and polyps. The correlation was the strongest in individuals > 30 years of age, for whom infection levels were low. Furthermore, a total of 62 out of 73 cytopathology Papanicolaoustained smears showed squamous metaplasia (Eissa *et al.*, 2007).

Regarding mutagenesis, in vitro studies did not succeed to show a mutagenic power of neither worm nor egg extracts (Osada *et al.*, 2005). On the whole, nitric oxide produced by the inflammatory response provoked by *S. haematobium* eggs, and alkylation of DNA by *N*-nitroso compounds did emerge as important causal factors of the DNA severe alterations related with *S. haematobium*-associated urinary bladder cancer (IARC, 2011).

### *Schistosoma Mansoni* and *S. Japonicum* (Digenea: Schistosomatidae)

Although there are not a clear association between cancer and *S. mansoni* infection geographical areas, *S. mansoni* parasitism was linked with liver, colorectal cancer and prostate cancer (Vennervald & Polman, 2009). Moreover, Brazilian pathologists have found giant follicular lymphomas in 1577 spleens removed from schistosomiasis mansoni patients with severe portal hypertension (Andrade et al., 1971; de Andrade *et al.*, 1982). The explanation of such observations remains unclear. Patients with schistosomiasis mansoni showed decreased hepatic levels of cytochrome p450, cytochrome b-5 and NADPH cytochrome C reductase. Such alterations could cause metabolites of aflatoxin to increase significantly, suggesting that *S. mansoni* could potentiate the effect of carcinogens (Habib *et al.*, 2006; Vennervald & Polman, 2009).

The association between *S. mansoni* infection and hepato-cellular carcinoma (HCC) could also be indirect. Patients with *S. mansoni* have higher incidence of HBV or HCV infection (two viruses associated with cancerogenesis) than noninfected controls (Halim *et al.*, 1999). The high exposure of schistosomiasis patients to HBV and HCV could be partly explained by transmission of these viruses during blood transfusion (via contaminated blood, syringes and needles) consecutive to hematemesis, which is a relatively frequent complication of hepatosplenic schistosomiasis, (Darwish *et al.*, 1993).

Regarding *S. japonicum*, its role in cancer occurrence is less clear, although this parasite has been associated with both liver and colorectal cancer. Some epidemiological and clinical studies in China and Japan support its role as one of the risk factors in HCC formation (Khurana *et al.*, 2005). Several epidemiological surveys have indicated a geographical association between *S. japonicum* infection and cancer of the liver and colon, but many of these studies did not consider other factors such as viral hepatitis, aflatoxin and other dietary carcinogens. On the whole, though an association between colon cancer and *S. japonicum* has been suggested as early as in the 60’, there has been no consistent evidence of *S. japonicum* as a carcinogen in humans.

### *Opisthorchis Viverrini* and *Clonorchis Sinensis* (Digenea: Opisthorchiidae)

*C. sinensis*, *O. viverrini* and *O. felineus* are closely related fish-borne zoonotic Digenetic trematodes, which infect humans and various fish-eating animals. *C. sinensis* and *O. viverrini* remain an important public health problem in many endemic areas in South-East Asia where they infect more than 20 million of persons (Khurana *et al.*, 2005). Indeed about 15 million people are infected with *C. sinensis* (rural areas of the Republic of Korea, Democratic People’s Republic of Korea, and China) and eight million with *O. viverrini* (Southeast Asian countries such as Thailand, Laos, Viet Nam and Cambodia) (Vennervald & Polman, 2009). Opisthorchiidoses due to infections with these two species were also reported in immigrants living in developed countries for many years (Attwood & Chou, 1978; Schmidt *et al.*, 1982; Vernes *et al.*, 1982). Actually, these fishborne flukes can persist for longtime in the host liver: life expectance of *O. viverrini* and *C. sinensis* are about ten years and 26 years, respectively (Attwood & Chou, 1978; Sithithaworn & Haswell-Elkins, 2003).

In addition to their association with hepatobiliary disease, these liver flukes are considered as major etiological agents of cholangiocarcinoma (CCA) (Parkin, 2006). After evaluating epidemiological studies, case series, and case control studies, *O. viverrini* and *C. sinensis* were considered definitely carcinogenic by the IARC (2011). Thus, nowadays, hepatobiliary opisthorchiasis is the strongest link which could be occurred between a human malignancy and a Metazoan infectious agent (Laha *et al.*, 2007).

The clinical manifestations, imaging findings and pathophysiology for *O. viverrini* and *C. sinensis* infection are almost the same (Lun *et al.*, 2005; Sripa *et al.*, 2009; Sripa & Pairojkul, 2008). Vennervald & Polman (2009) have reviewed recently these aspects. The adult liver flukes are found in the biliary system, especially in the medium-sized or small intrahepatic bile ducts causing mechanical obstruction, inflammation, adenomatous hyperplasia and periductal fibrosis. Thus, in contrast with Fasciola hepatica, the Opisthorchiidae worms do not invade the hepatic parenchyma and hence, most of the disease manifestations result from the mechanical irritation and associated pathological changes caused by the worms to the biliary epithelium. Extensive and chronic infections can cause severe hepatobiliary disease manifestations such as cholangitis, cholecystitis, cholelithiasis, obstruction jaundice, hepatomegaly and fibrosis of the periportal tract (Vennervald & Polman, 2009). Worldwide, CCA (derived from the epithelial cells or cholangiocytes lining the bile ducts) is much less common than hepatocellular carcinoma (HCC, derived from hepatocytes), except for those areas in East Asia, where infection with *O. viverrini* or *C. sinensis* is widespread and highly prevalent in humans (Srivatanakul *et al.*, 2004; Porta *et al.*, 2011).

The mechanisms of carcinogenesis due to liver fluke infection remain unclear. *N*-nitroso-compounds are generated locally from NO produced by inflammatory cells around the bile ducts. NO may lead to DNA damage, particularly in proliferating bile duct cells (Sripa *et al.*, 2007). Furthermore, recent evidence suggests that *O. viverrini* secretes mitogenic proteins in host tissues where they might promote cell proliferation, mutagenesis and finally carcinogenesis (Laha *et al.*, 2007; Sripa *et al.*, 2007). Moreover, the flukes induced *in vitro* over-expression of mRNAs encoding growth-promoting proteins including transforming growth factor (Thuwajit *et al.*, 2006). Regarding *C. sinensis*, this worm releases products that induce cell proliferation, associated with up-regulation of cyclin B and transcription factor E2F1 (Kim *et al.*, 2008). Despite the direct effect of liver fluke molecules on cell proliferation, liver fluke-induced CCA results likely from a slow indirect process closely linked to chronic inflammation that involves the activation of oxidative stress pathways (Sripa *et al.*, 2007).

## Conclusion

Some causal associations between parasites and cancers have been established already in humans and in experimental animals. More research in the field is warranted to substantiate additional links. In addition to clinical and epidemiological data, molecular factors or mechanisms linking parasite infection and cancer development need still to be investigated, especially, host chronic inflammatory response, which is a widely recognized basic mechanism for the development of most of infection-related tumors.

Developing strategies designed to prevent or appropriately treat cancer-associated parasites arise as a major issue of Public Health, especially among the bottom billion living in the poorest areas of the developing world. In these areas, where parasitic infections are highly prevalent, the underlying causes of parasiteassociated cancers can be frequently neglected. Avoiding exposure, reducing parasite transmission, and treating infection early could implement this prevention potential, reducing the global impact of some cancers, mainly but not only in developing countries. These strategies must, however, be built on sound scientific evidence.

On the other hand, in many cases, parasitic agents are widely present in humans, but only a small fraction of infected individuals develop cancer. Thus, it is also important to identify subpopulations of exposed individuals who are likely to develop cancer by developing sensitive and specific molecular markers in order to be able to implement preventive strategies. Together, parasitic determinants of cancer offer a spectrum of research and prevention possibilities that could substantially affect global health by reducing some cancers worldwide.
